# Supplementary material for: Effects of Surfactants on Zein Cast Films for Simultaneous Delivery of Two Hydrophilic Active Components
Source: Materials (Basel). 2022 Apr 11;15(8):2795. doi: 10.3390/ma15082795 (PMC9027419; doi:10.3390/ma15082795)
Supplement: Supplementary file 1 [file materials-15-02795-s001.zip › materials-1660005-Supplementary.pdf]

materials

Supplementary

Effects of Surfactants on Zein Cast Films for Simultaneous Delivery of Two Hydrophilic Active Components

Dongwei Wei [1], Fanhui Zhou [1], Hongdi Wang [2], Guijin Liu [3], Jun Fang [1,*] and Yanbin Jiang [4,*]

[1] School of Chemical Engineering and Materials Science, Quanzhou Normal University, Quanzhou 362000, China; wdw2017@qztc.edu.cn (D.W.); jingxu@qztc.edu.cn (F.Z.)
[2] Key Laboratory of Organosilicon Chemistry and Material Technology, College of Material, Chemistry and Chemical Engineering, Ministry of Education, Hangzhou Normal University, Hangzhou, 311121, China; wang.hongdi@hznu.edu.cn
[3] School of Pharmaceutical Sciences, Hainan University, Haikou 570228, China
[4] School of Chemistry and Chemical Engineering, South China University of Technology, Guangzhou 510640, China
* Correspondence: fangjun@qztc.edu.cn (J.F.); cebjiang@scut.edu.cn (Y.J.)

Table S1. Kinetic parameters determined from release curves of LY of the cast films.

Films	Initial LY Release Rate ($U/(cm^2$ h))	Total Released LY (U/cm^2)
2	11920 ± 402 a $^{a\ b}$	41574 ± 458 a
3	579 ± 25 g	1296 ± 60 h
4	228 ± 10 g	1375 ± 69 h
5	2834 ± 70 ef	2682 ± 123 g
6	6968 ± 230 bc	19805 ± 720 d
7	4152 ± 153 de	23477 ± 1102 b
8	5326 ± 182 cd	18280 ± 596 ef
9	7005 ± 220 bc	21075 ± 860 c
10	4497 ± 206 de	22971 ± 1140 b
11	8417 ± 189 ab	19225 ± 820 de
12	352 ± 14 g	1423 ± 71 h
13	3205 ± 124 def	-
14	1398 ± 43 fg	17567 ± 532 f

[a] Determined from the slope of the initial linear portion of release curves. r^2 values of curves were between 0.5022 and 0.8625. [b] Different letters in each column show significant difference $P < 0.05$.

Table S2. Kinetic parameters determined from release curves of AA and anti-oxidant activity of films.

Films	Initial AA Release Rate ($mg/(cm^2$ h))	Total Released AA (mg/cm^2)	Anti-oxidant Activity (μmol $Trolox/cm^2$)
2	2.23 ± 0.07 b $^{a\ b}$	2.63 ± 0.13 a	151.9 ± 2.9 g
3	1.96 ± 0.07 c	2.27 ± 0.11 cd	138.0 ± 1.8 h
4	1.85 ± 0.06 cd	1.94 ± 0.09 e	86.2 ± 0.9 i
5	2.17 ± 0.08 b	2.42 ± 0.11 bc	177.5 ± 2.6 e
6	1.48 ± 0.05 gh	2.03 ± 0.08 e	216.3 ± 2.1 b
7	1.34 ± 0.04 ij	1.88 ± 0.09 e	204.8 ± 3.6 c
8	1.25 ± 0.06 j	1.43 ± 0.07 f	163.4 ± 0.7 f
9	1.70 ± 0.05 ef	2.44 ± 0.12 bc	180.2 ± 1.9 e
10	1.66 ± 0.09 ef	2.36 ± 0.10 bcd	178.1 ± 1.8 e
11	1.58 ± 0.04 fg	2.33 ± 0.10 bcd	176.4 ± 2.1 e
12	1.76 ± 0.05 de	2.47 ± 0.13 ab	185.4 ± 1.9 d
13	2.40 ± 0.08 a	-	-
14	1.41 ± 0.12 hi	2.22 ± 0.31 d	226.0 ± 3.2 a

[a] Time periods (h) of data used in best fitting curves were between 0 and 1 h. r^2 values of curves were between 0.5465 and 0.9836. [b] Different letters in each column show significant difference $P < 0.05$.

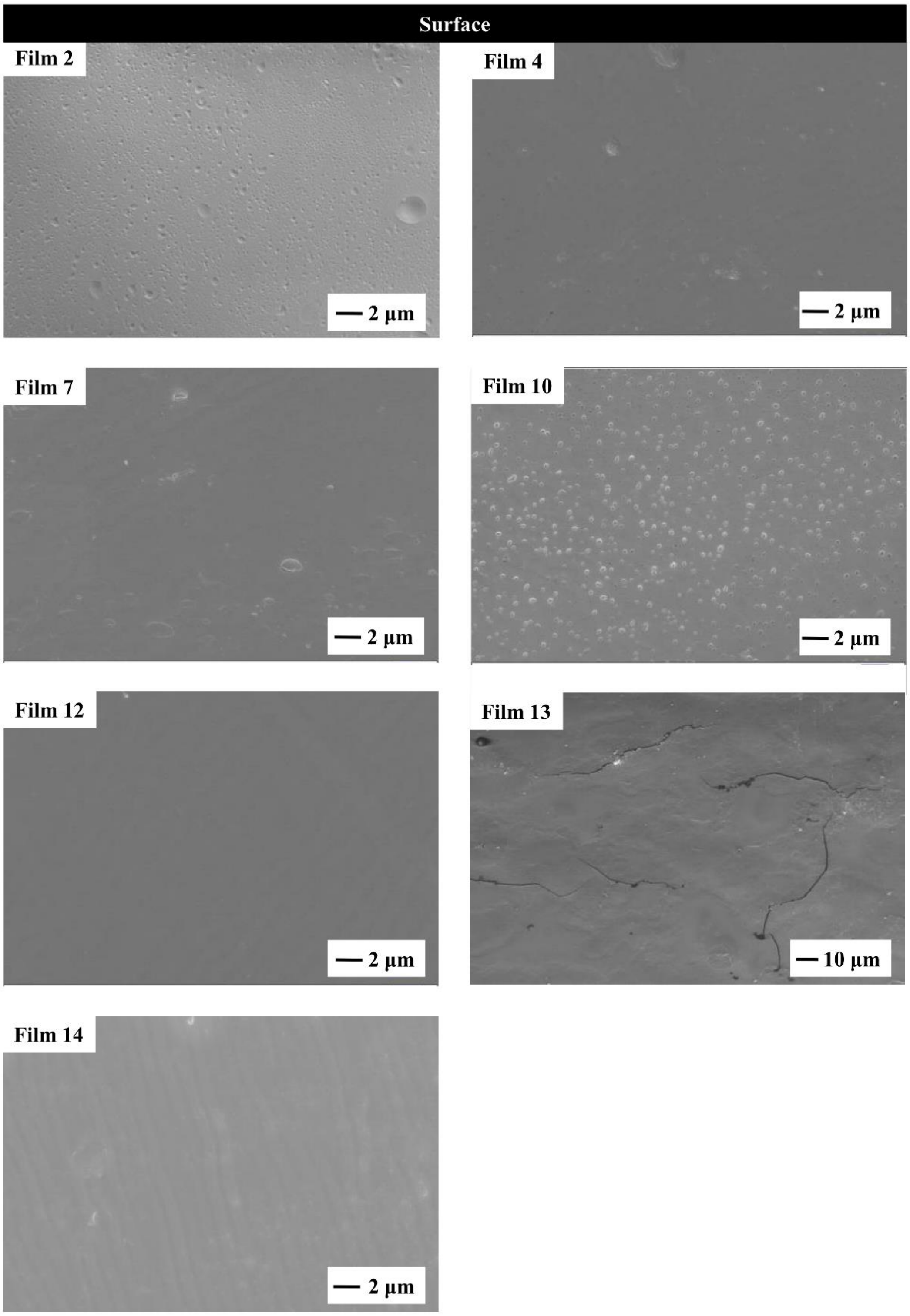

Figure S1. Surface SEM images of prepared zein cast films.

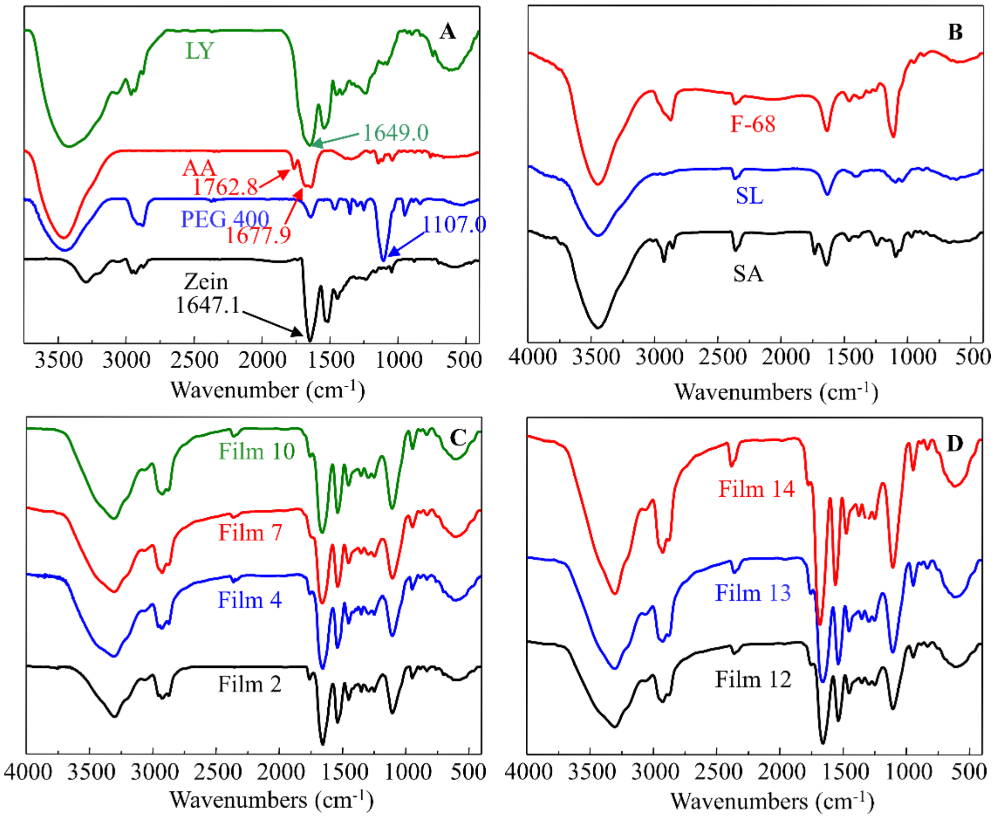

Figure S2. FT-IR of LY, AA, PEG 400 and zein (**A**); SA, SL and PF-68 (**B**) and the developed typical zein cast films (**C** and **D**).
